# Hepatitis B virus core promoter mutations G1613A and C1653T are significantly associated with hepatocellular carcinoma in genotype C HBV-infected patients

**DOI:** 10.1186/1471-2407-11-458

**Published:** 2011-10-21

**Authors:** Masashi Tatsukawa, Akinobu Takaki, Hidenori Shiraha, Kazuko Koike, Yoshiaki Iwasaki, Haruhiko Kobashi, Shin-Ichi Fujioka, Kohsaku Sakaguchi, Kazuhide Yamamoto

**Affiliations:** 1 Department of Gastroenterology and Hepatology, Okayama University Graduate School of Medicine, Dentistry, and Pharmaceutical Sciences, Okayama, Japan

## Abstract

**Background:**

Hepatitis B virus (HBV) is a major cause of hepatocarcinogenesis.

To identify mutations relevant to hepatocellular carcinoma (HCC) development, we compared the full genome sequences of HBV from the sera of patients with and without HCC.

**Methods:**

We compared the full genome sequences of HBV isolates from 37 HCC patients (HCC group 1) and 38 patients without HCC (non-HCC group 1). We also investigated part of the core promoter region sequences from 40 HCC patients (HCC group 2) and 68 patients without HCC. Of the 68 patients who initially did not have HCC, 52 patients remained HCC-free during the follow-up period (non-HCC group 2), and 16 patients eventually developed HCC (pre-HCC group 2). Serum samples collected from patients were subjected to PCR, and the HBV DNA was directly sequenced.

**Results:**

All patients had genotype C. A comparison of the nucleotide sequences of the HBV genome between HCC group 1 and non-HCC group 1 revealed that the prevalence of G1613A and C1653T mutations in the core promoter region was significantly higher in the HCC group. These mutations tended to occur simultaneously in HCC patients. Multivariate analysis with group 2 revealed that the presence of HCC was associated with aging and the double mutation. Future emergence of HCC was associated with aging and the presence of a single G1613A mutation.

**Conclusions:**

G1613A and C1653T double mutations were frequently found in patients with HCC. A single G1613A mutation was associated with future emergence of HCC. These mutations may serve as useful markers in predicting HCC development.

## Background

Approximately 2 billion people worldwide have been infected with the hepatitis B virus (HBV). Of these, 350 to 400 million are chronic carriers of the virus [1,2]. The clinical state of HBV infection is variable, ranging from acute hepatitis to various forms of chronic liver disease, such as chronic hepatitis, cirrhosis and hepatocellular carcinoma (HCC) [1].

Chronic HBV infection is a risk factor for HCC. Chronic inflammation and liver fibrosis caused by chronic HBV infection contributes to the occurrence of HCC [1]. However, in addition to these host factors, HBV itself has a direct role in the development of HCC [3,4]. A significant proportion of HBV-related HCCs arise in otherwise normal livers [5,6], and animal models transfected with this virus genome develop HCC; these observations confirm the oncogenic potential of HBV in the liver [3,7]. Although a considerable amount of research has been conducted, the molecular basis of HBV-related hepatocarcinogenesis remains unknown [8,9].

If HBV were directly oncogenic, variations in HBV could account for some of the heterogeneity in the clinical course of HCC development. Several studies have examined mutations within the HBV genome that may be associated with HCC. Genotypic diversity is related to differences in clinical and virological characteristics. For example, genotype C infected patients have more severe chronic liver disease, including cirrhosis and HCC [5,10]. As for deletions and point mutations, which are more subtle genetic variations than genotype, previous studies have found some clues, such as codon 38 in × gene, A1762T/G1764A basal core promoter (BCP) mutation, and deletion of pre-S and × protein [11-13], but definitive conclusions have not yet been made.

Bias might be created by studying a limited number of patients, a limited section of the HBV-DNA sequence, and patients with different genotypes of HBV. Therefore, whole sequences of HBV-DNA from a genetically similar cohort of patients infected with the same HBV genotype might reduce viral genotypic and host genetic biases. In the present study, we investigated whole genome sequences of circulating HBV-DNA from the sera of HCC patients who were infected with HBV genotype C in the western part of Japan.

## Methods

### Patients

Between 1988 and 2004, a total of 575 patients in our hospital were diagnosed as having HBV-related chronic liver diseases, and 138 of these patients developed HCC. Seventy-five patients were diagnosed with HCC at their first visit, and 63 patients were diagnosed with HCC during the subsequent follow-up period. The patient age range was from 29 to 75 years old, and there were 27 women and 111 men.

We excluded patients who were presumed to have acute hepatitis B based on their sexual history, intravenous drug use or exposure to blood products, serological markers including high IgM-HBc antibody positivity and imaging findings showing no chronic liver disease with ultrasonography or computed tomography. Patients who had a history of alcoholism or other causes of hepatitis, such as autoimmune hepatitis, primary biliary cirrhosis, and Wilson's disease were also excluded. We also excluded patients with a very low viral load due to our inability to extract HBV DNA from their serum. In total, we studied 217 serum samples from 183 patients with chronic HBV. Informed consent was obtained from each participant. The study was approved by the ethics committee of Okayama University Hospital.

Diagnosis of HCC was based on positive findings on ultrasonography, computed tomography and/or angiography.

### Design of the study

The overall study consisted of the following: (1) a matched case-control study was designed to find HBV mutations (from the entire HBV genome) significantly associated with HCC; (2) an unmatched case-control study was designed to validate the mutations associated with HCC; and (3) a nested case-control study in a cohort designed to see if the mutations found in the previous two case-control studies at baseline can predict the occurrence of HCC in patients with chronic hepatitis B.

### Isolates used for complete genome sequencing

Complete viral genome sequencing was performed on viral DNA isolated from the sera of 37 patients who had HCC (HCC group 1) and 38 patients who did not have HCC or develop HCC during the follow-up period (non-HCC group 1). The mean follow-up period was 8.1 years. Serum samples from patients in non-HCC group 1 were collected at their first visit. The ages and sex of non-HCC group 1 patients were matched with HCC group 1 patients. The patients from HCC group 1 and non-HCC group 1 had an HBV titer that was sufficient to extract enough DNA to determine the entire nucleotide sequence of the HBV genome. Patients did not have on nucleos(t)ide analogue treatment at the time of serum correction to extract enough DNA for sequencing. Liver histology was available for only 12 patients in non-HCC group 1 and was not used for statistical analysis. Three patients had stage 1 fibrosis; 6 patients had stage 2 fibrosis; and 3 patients had stage 3 fibrosis.

The characteristics of these patients are shown in Table 1. We could not calculate the MELD (Model for End-Stage Liver Disease) score as a liver function marker because prothrombin time data for many patients were unavailable. We compared the sequences of the entire HBV genome between the two groups.

**Table 1:** Clinical parameters of the subjects used for HBV sequencing

whole HBV sequencing			
	HCC group 1	non-HCC group 1	
	(n = 37)	(n = 38)	
	
Age (Years)	37 ± 10.0	38 ± 11.6	
Sex:Male	29 (78%)	28 (73%)	
HBV-DNA (log/ml)	5.5 ± 1.1	5.2 ± 0.9	
HBeAg positive	20 (54%)	20 (52%)	
Platelet (× 10^4^/μl)	11.4 ± 5.3	16.4 ± 6.0	
Total Bilirubin (mg/dl)	1.3 ± 1.1	1.6 ± 1.97	
Albumin (g/dl)	3.58 ± 0.8	4.0 ± 0.44	
	
core promoter sequencing			

	HCC group 2	non-HCC group 2	pre-HCC group 2
	(n = 40)	(n = 52)	(n = 16)

Age (Years)	54 ± 13.1	38 ± 11.4*#	48 ± 10.2
Sex:Male	78%	83%	94% *
HBV-DNA (log/ml)	5.76 ± 2.04	7.23 ± 1.37*	6.42 ± 1.66
HBeAg positive	55%	75%	79%
Platelet (× 10^4^/μl)	12.1 ± 6.39	17.1 ± 7.15*	15.0 ± 6.59
Total Bilirubin (mg/dl)	1.32 ± 1.13	1.29 ± 2.07	2.55 ± 6.66
Albumin (g/dl)	3.58 ± 0.84	4.10 ± 0.51*	3.84 ± 0.57

*p < 0.05 vs. HCC group 2, #p < 0.05 vs. pre-HCC group 2

### Isolates used for core promoter sequences

To define the reliability of correlations between core promoter mutations and hepatocarcinogenesis drawn by the whole genome analysis, we sequenced a small part of the core promoter region of an additional 108 isolates. Forty isolates were obtained from the HCC patients (HCC group 2), and 68 isolates were from the patients without HCC at their first visit. Of these 68 patients, 16 patients who developed HCC during the follow-up period were defined as pre-HCC group 2, and 52 patients who did not develop HCC were labeled as non-HCC group 2.

The mean follow-up period was 5.8 years for the non-HCC group 2 and 9.5 years for the pre-HCC group 2.

### Extraction and amplification of serum HBV DNA

Serum samples were stored at -30°C until use. Nucleic acids were extracted from 200 μL of serum using a commercially available kit (QIAamp DNA Blood Mini Kit; Qiagen Inc., Valencia, CA).

To obtain a full-length HBV DNA sequence from the nucleic acids, we performed nested PCR to amplify two overlapping fragments with a PfuTurbo polymerase kit (Stratagene, La Jolla, CA). The PCR primer sequences and the relative positions of these PCR fragments on the genetic map of HBV DNA are shown in Table 2.

**Table 2:** Primers used sequencing

Primer	Nucleotide Sequence (5' 3')	Position	
HB1F	AAGCTCTGCTAGATCCCAGAGT	18-39	Sense
HB1R	GAAACATAGAGGTGCCTTGAGCAG	557-534	Antisense
HB2F	TGCTGCTATGCCTCATCTTC	414-433	Sense
HB2R	CATACTTTCCAATCAATAGG	989-970	Antisense
HB3F	GCCAAGTCTGTACAACATCTTGAG	760-783	Sense
HB3R	AGTTGGCGAGAAAGTGAAAGCCTG	1107-1084	Antisense
HB4F	CCTATTGATTGGAAAGTATGTCA	970-992	Sense
HB4R	CGGGACGTAGACAAAGGACGT	1434-1414	Antisense
HB5F	CTCTGCCGATCCATACTGCGGAA	1256-1278	Sense
HB5R	TTAACCTAATCTCCTCCCCCA	1761-1741	Antisense
HB6F	TTGTYTACGTCCCGTCGGCG	1421-1440	Sense
HB6R	AACAGACCAATTTATGCCTA	1803-1784	Antisense
HB7F	GAGACCACCGTGAACGCCCA	1611-1630	Sense
HB7R	CCTGAGTGCTGTATGGTGAGG	2072-2048	Antisense
HB8F	TTCACCTCTGCCTAATCATC	1824-1843	Sense
HB8R	ATAGGGGCATTTGGTGGTCT	2314-2278	Antisense
HB9F	TCAGGCAACTATTGTGGTTTCA	2190-2211	Sense
HB9R	GGATAGAACCTAGCAGGCAT	2654-2635	Antisense
HB10F	CGCAGAAGATCTCAATCTCGG	2417-2437	Sense
HB10R	GGGTTGAAGTCCCAATCTGGATT	2987-2965	Antisense
HB11F	GGGTCACCATATTCTTGGGAA	2814-2834	Sense
HB11R	GAACTGGAGCCACCAGCAGG	75-56	Antisense
HB12F	GTGGAGCCCTCAGGCTCAGG	3075-3094	Sense
HB12R	CGAGTCTAGACTCTGTGGTA	256-237	Antisense

For sequencing a small part of the core promoter region, we performed PCR using 5F/5R primers

### Nucleotide sequencing of PCR products

PCR products were sequenced directly by the dideoxy termination method using the Big Dye Terminator cycle sequencing Ready Reaction Kit ver. 3.1 (Applied Biosystems, Foster City, CA) with modifications and an automatic sequencer (ABI PRISM 3100 Genetic Analyser; Applied Biosystems). PCR products were sequenced in forward and reverse directions. Sequence alignment was performed by using the multiple-alignment algorithm in MegAlign (DNASTAR, Windows version 5.06, WI).

### Statistics

SAS software (Version 6, SAS Institute Inc., NC) was used to conduct the statistical analysis. Continuous data were expressed as mean ± SD and were analyzed with one-way ANOVA for normally distributed data and with the Kruskal-Wallis test for other data. Categorical data were analyzed with Pearson's Chi-square test. If a statistical difference was found, we compared the groups by using a Chi-square test with Bonferroni's correction. Logistic regression analysis was used to assess the likelihood of a nucleotide change due to the influence of various factors related to the risk of HCC development. Cumulative HCC incidence curves were determined using the Kaplan-Meier method and the differences between groups were assessed with the log-rank test. Cox proportional hazard regression analysis was used to identify significant factors that influence future HCC development.

## Results

### Genotyping

Phylogenetic trees that were constructed using the neighbor-joining method revealed that all patients in our study had genotype C and subgenotype C2.

### Analysis of full sequences

To identify mutations associated with hepatocarcinogenesis, we studied the complete nucleotide sequences of the HBV genome isolated from the sera of patients who had HCC (HCC group 1; [DDBJ: AB670237-AB670273]) and who had not developed HCC (non-HCC group 1; [DDBJ: AB670274-AB670311]).

Several deletions were found in the HBV genome. Most deletions were located in the pre-S and × regions. However, there were no significant differences between HCC group 1 (2 patients with pre-S1 deletions, 1 patient with pre-S2 deletion) and non-HCC group 1 (2 patients with pre-S1 deletions, 3 patients with BCP/X deletions).

To identify point mutations relevant to HCC, we compared each nucleotide of the whole sequences of the HBV genome between HCC group 1 and non-HCC group 1. Of all 3215 base pairs, only two nucleotides showed significant differences between HCC group 1 and non-HCC group 1 (Table 3). The two mutations were both located in the core promoter region. A G-to-A mutation at nucleotide 1613 occurred in 38% of HCC group 1 and 10% of non-HCC group 1 (p < 0.008). A C-to-T mutation at nucleotide 1653 occurred in 45% of HCC group 1 and 19% of non-HCC group 1 (p < 0.05).

**Table 3:** The prevalence of mutations in the samples

	Mutation frequency		Locations in open reading frames			
	**HCC group 1**	**non-HCC group1**	**Pol**	**X**	**C**	**S**

G162A	42%	26%	357			177
A306G	9%	0%	405			225
A456G	10%	0%	455			275
C928T	37%	40%	613			
C955T	11%	8%	622			
A1032C	0%	7%	647			
A1053G	33%	19%	654			
A1126C	36%	41%	679			
T1134C	36%	35%	681			
T1323C	9%	16%	744			
G1356A	7%	0%	755			
C1485T	25%	27%	798	38		
G1499A	37%	34%	803	42		
G1511A	29%	23%	807	46		
G1613A	38%	10%*	841	80		
A1633G	5%	0%		87		
C1653T	45%	19%*		94		
G1727A	36%	51%		118		
A1762T	74%	77%		130		
G1764A	80%	84%		131		
G2080A	13%	14%			89	
C2444A	10%	0%	46		211	
A2574G	24%	14%	90			
C2586A	7%	0%	94			
A2696G	8%	0%	130			
G2783A	38%	33%	159			
C2919T	5%	0%	205			24
C3026T	20%	16%	240			60
T3098C	19%	2%	84			264

*p < 0.05 vs. HCC group 1

The A1762T/G1764A BCP mutation rate was not different between HCC group 1 and non-HCC group 1 (75% vs. 72%). However, the BCP mutation was frequently found in conjunction with the G1613A mutation or the C1653T mutation. The odds ratio of the BCP mutation was 8.2 in the G1613A mutant and 4.62 in the C1653T mutant as compared to wild type (p < 0.05).

### Comparison of sequences in the core promoter region

We suspected that the two mutations in the core promoter region were relevant to hepatocarcinogenesis. To determine the reliability of this result, we examined an additional 108 isolates to sequence a part of the core promoter region. The results of the core promoter region analysis were similar to our results from the whole genome sequencing. The rate of the G1613A and C1653T mutations were significantly higher in patients with HCC (Table 4). The rate of the G1613A and C1653T mutations was 50% and 50% in HCC group 2, while each of these mutations was found in only 12% of the non-HCC group 2 (p < 0.05). Statistical analysis revealed that G1613A and C1653T mutations were dependent on each other. The rate of the G1613A mutation was significantly higher in isolates with the C1653T mutation as compared to isolates without the C1653T mutation (65% vs. 34%, p < 0.05) and vice versa (65% vs. 34%, p < 0.05). The combination of these mutations was significant in HCC group 2. The G1613A and/or the C1653T mutations were found at a significantly lower frequency in the non-HCC group 2. Clinical parameters, such as aging, albumin level and platelet count, showed a tendency to be related to HCC group 2 (Table 1), indicating that these mutations might occur with liver fibrosis progression. However, the double mutation rate was high only in HCC group 2 (Table 4).

**Table 4:** The frequency of G1613A and C1653T mutations

	HCC group 2	non-HCC group 2	pre-HCC group 2
	(n = 40)	(n = 52)	(n = 16)
*mutation*			
G1613A	50%	12%* #	38% *
C1653T	50%	12%*	13% *
G1613A and C1653T	35%	6%*	0%*
G1613A and/or C1653T	65%	17%* #	50%

*p < 0.05 vs. HCC group 2, #p < 0.05 vs. pre-HCC group,

### Patient characteristics and the 1613/1653 mutations

We determined whether the positivity of the HBe antigen (HBeAg) was related to the mutations. The G1613A mutation was found in 32% of HBeAg-positive patients and 24% of HBeAg-negative patients (N.S. data not shown). The C1653T mutation was found in 27% of HBeAg-positive patients and in 31% of HBeAg-negative patients (N.S.). The double mutation rate was not different with positivity of the HBeAg (14% HBeAg-positive vs. 18% HBeAg-negative). The positive rate of HBeAg did not correlate with these mutations (Table 5). The mutations tended to occur with aging in HCC group 2, although this tendency was not statistically significant. The ratio of mutation was not different with aging in non-HCC group 2.

**Table 5:** Mutations and clinical findings

HCC group 2 (n = 40)				
Mutation (1613/1653)	-/-	+/-	-/+	+/+

n	14	6	6	14

HBeAg (positive)	40%	60%	50%	67%
Sex (Male)	79%	83%	83%	71%
Patient age	54.6 ± 14.3-	43.3 ± 6.7	57.8 ± 18.3	57.8 ± 9.5

Non-HCC group 2 (n = 52)				

Mutation (1613/1653)	-/-	+/-	-/+	+/+

n	43	3	3	3

HBeAg (positive)	78%	50%	33%	100%
Sex (Male)	81%	100%	67%	100%
Patient age	37.8 ± 11.0	32.0 ± 17.3	48.7 ± 7.0	30.7 ± 10.2

### Multivariate analysis

To evaluate the effect of the mutations on the development of HCC, multiple logistic regression analysis was performed with the clinical features and the G1613A and C1653T mutations (Table 6).

**Table 6:** Multivariate analyses for the development of HCC

1.Factors associated with HCC presence			
	Multivariate analysis		
	
Factor	Odds ratio	95% CI	p value

Age (≥ 45)	7.13	2.39-23.39	0.0004
G1613A and C1653T	7.19	1.42-56.0	0.016

2. Factors associated with future HCC emergence			

	Multivariate analysis		
Factor	Odds ratio	95% CI	p value

Age (≥ 45)	4.89	1.41-18.69	0.010
G1613A	4.73	1.11-21.69	0.035

3. Factors associated with the occurrence of HCC by			
Multivariate Cox proportional hazard regression analysis			

Factor	Odds ratio	95% CI	p value

Platelet (< 14 × 10^4^/μl)	6.28	1.66-30.11	0.0065
Factor	Odds ratio	95% CI	p value
Platelet (< 14 × 10^4^/μl)	6.28	1.66-30.11	0.0065

In our first analysis, we used HCC group 2 and non-HCC group 2 to evaluate the effect of the double mutation on the presence of HCC. The following characteristics, which were proven to be statistically significant with univariate analysis, were used for multiple logistic regression analysis: age ≥45, Plt (×10^4^/μl) ≤14, ALT (≥70), and the presence of G1613A and C1653T. The presence of HCC was associated with the double mutation and increased age.

In our second analysis, we used non-HCC group 2 and the pre-HCC group 2 to evaluate the effect of the mutation on the later occurrence of HCC. The following characteristics, which were proven to be statistically significant with univariate analysis, were used for multiple logistic regression analysis: age ≥45 years, male, and the G1613A mutation. The presence of the G1613A mutation was significantly higher in the group that later developed HCC. However, cox proportional hazard regression analysis showed only low platelet count to be the predictor of later HCC occurrence.

## Discussion

In the present study, we compared the whole HBV genome sequence of 37 HCC patients with 38 non-HCC patients. All patients were infected with genotype C viruses, and the most common mutations were G1613A and C1653T. Patients with both mutations were at a particularly high risk for the presence of HCC. The association between these mutations and hepatocellular carcinoma development was confirmed in an analysis of 183 subjects. An analysis of HCC development during the patient follow-up period revealed that the presence of G1613A and/or C1653T mutations was more prevalent in patients who developed HCC long afterward, as compared to the patients who never developed HCC.

Several studies have revealed the relationship between mutations and hepatocarcinogenesis [9,13-18]. Our hospital is located in the western side of Japan, where genotype C is endemic [19], population mobility is low and the ethnicity is homogeneous. The resulting similarities among study subjects support the reliability of our results.

The risk for hepatocarcinogenesis is higher in genotype C HBV patients as compared with other genotypes [20]. In different genotypes, the effect of a single point mutation on the function of viral proteins could be different. Our results shed light on the risk of viral mutations on hepatocarcinogenesis in high-risk HBV genotype C (subgenotype C2)-infected patients.

The G1613A and C1653T mutations are both located in the core promoter region [21]. Mutations in the core promoter region have clinical consequences to the host caused by changes in viral characteristics, such as transcription of the pregenomic and pre-C RNAs.

The nucleotide at position 1613 is located at the N-terminal end of the negative regulatory element, upstream of Enhancer II (ENII), which can repress the function of ENII [22]. Mutational analysis has shown that the sequence from 1612 to 1615 is responsible for the repression activity of the negative regulatory element [23]. The 1613 mutation can result in the failure of the repression function of the negative regulatory element and in the proliferation of virus-related proteins that might induce hepatocarcinogenesis. Nucleotide 1613 is also located within overlapping open reading frames, the × gene at codon 80 (which results in a synonymous change in × protein) and polymerase gene, which contains the C-terminal side of the RNaseH domain (A to K change).

The nucleotide at position 1653 is located in the core upstream regulatory sequence (CURS) region, which has a strong stimulatory effect on the basic core promoter independent of enhancer II [24]. CURS is divided into two regions, A (5'-half; nucleotides 1636-1703) and B (3'-half; nucleotides 1704-1743), which can independently stimulate BCP activity. The 1653 position is located in the box alpha domain that might change the stimulatory effect on BCP. Nucleotide 1653 is also located within the × gene at codon 94. × protein binds and affects several intracellular signal transduction pathways, cell cycle progression, and apoptosis regulation [25]. Codon 94 of × protein is included in area of several transcription factor binding sites, such as CREB, RPB5, TFIIB, XAP-1, C/EBPalpha, and XAP-3. Although this region does not directly bind p53 protein, it contains a p53-dependent transcriptional repression binding site that is necessary for negative regulation of a p53-induced viral-specific DNA enhancer [21,26].

Several previous studies have reported the association of the mutations at 1613 or 1653 with HCC development. In a Japanese study, Takahashi et al. found that G1613A and C1653T occurred in 38% and 40%, respectively, of 40 HCC patients [15]. However, their study did not include a non-HCC control group and did not analyze confounding host factors. Shinkai et al. reported that the combination of C1653T and/or V1753 mutations in addition to T1762/A1764 mutations was differentially associated with HCC in HBeAg-positive genotype C patients [27]. Although nucleotide 1613 was outside the range of their sequence analysis, their data were partially consistent with our observations. A recent study from China revealed that 1613 and 1653 mutations were included in mutations frequently detected in HCC patients; however, core region mutants A2189C and G2203W were independent risk factors for HCC [18]. A meta-analysis of 43 studies revealed that PreS mutation, C1653T, T1753V, and A1762T/G1764A were dominant in HCC [28]. PreS mutation in genotype C virus was more strongly associated with HCC than PreS mutation in genotype B virus. We could not find any significant differences of the PreS region. Our patient population had an average age of 38 years, which might be too young to accumulate such mutations or large deletions.

Although there have been many reports of important mutations, a definite consensus regarding the significance of mutations in HCC development has not yet been reached. Several factors have made it difficult to reach a consensus. It is difficult to define non-HCC patients, and even when patients are HCC-free for several years, they may eventually develop HCC. Moreover, there are many confounding factors in hepatocarcinogenesis, such as patient age, HBeAg status, and liver cirrhosis. Another limitation of previous studies was the relatively small number of subjects (40 or less) and the mixture of several HBV genotypes. We compared 37 HCC patients with 38 non-HCC patients. Our multivariate analysis with 40 HCC group 2 patients and 52 non-HCC group 2 patients revealed that HCC was associated with increased age and the presence of the 1613/1653 double mutation. The mutations might increase with aging; however, the age matched case-control study revealed the significance of the mutations. The future emergence of HCC is correlated with only G1613A mutation, while the double mutation was associated with the presence of HCC. Acquiring the double mutation might affect the carcinogenesis. Further studies regarding the functional changes in HBV DNA induced by this combination of mutations are clearly needed.

The BCP or precore mutations have been reported to be associated with patient characteristics, such as age, sex, HBeAg-positive status and the clinical course as fulminant hepatitis and liver cirrhosis, especially in genotype C [21,29-31]. Since these mutations are frequently found among patients without HCC, the use of these mutations as a marker for HCC surveillance may not be cost effective [32]. In the present study, the BCP mutation was found in 75% of HCC patients and 72% of non-HCC patients, which is not statistically different. Since our subjects all had genotype C virus, this high frequent BCP mutation rate is appropriate. In genotype C virus infected patients, the BCP mutation is reported to accumulate as aging and associated with an increased risk of HCC, however, when the BCP mutation coexisted with precore mutation, the risk of HCC have decreased [33,34]. Probably the BCP mutation needs combination of other several mutations to induce hepatocarcinogenesis. Our patients' ages were 37 for HCC group 1 and 38 for non-HCC group 1 suggesting young enough not to accumulate these mutations. The G1613A and C1653T mutations were not associated with sex or HBeAg-positive status. Our follow-up analysis revealed that the G1613A mutation was prevalent in patients who would later develop HCC. This result suggests that these mutations could predict HCC development. In our study, the mean follow-up period was 8.1 years. Although this amount of time is not a measure of the whole lifetime of patients with chronic HBV infection, it is long enough to evaluate the incubation period of HCC.

The PreS mutation in genotype C virus is more strongly associated with HCC than the PreS mutations in genotype B virus [35]. Deletions of preS2 or × have been reported to be associated with HCC development [33,34]. However, in our study, deletions in the pre-S1/S2 gene were observed similarly in the HCC group and non-HCC group. Our patient population might be too young to accumulate such mutations or large deletions as BCP were. Pre-S2 variants have been revealed that could not express pre-S2 and normal sized pre-S1 proteins, resulting in the retention of HBs antigen (HBsAg) in the cytoplasm with a subsequent decrease in HBsAg and HBV-DNA in serum [34]. In the present study, we included only the subjects with high viral load that was sufficient to analyze the whole genome sequences. This may be one additional reason why we did not frequently find pre-S2 variants, which should be found in patients with a low viral load.

The predictive value of the mutation results is relatively high. This might be the results of small number of the cohort as the 95% confidence interval is wide. For more precise analysis, we need more number of the patients. We had attempted to add more samples for sequencing. However, recently diagnosed patients are almost all treated with nucleos(t)ide analogues. As a result, the viral DNA levels are very low, and it is difficult to add more samples.

## Conclusions

We analyzed the full-length sequence of peripheral blood serum HBV DNA of HCC and non-HCC patients. Two mutations in the core promoter region, G1613A and C1653T, were associated with hepatocarcinogenesis. A single G1613A mutation was associated with future emergence of HCC that could be a marker for predicting HCC development.

## Competing interests

The authors declare that they have no competing interests.

## Authors' contributions

MT carried out the all sequences and clinical data consumption. AT participated the statistical analysis and drafted the manuscript. KK participated the sequence analysis. HS participated the sequence technique. YI participated clinical data consumption.

HK participated clinical data consumption. SF participated clinical data consumption. KS participated clinical data consumrtion. KY participated clinical data consumption and analysis and interpretation of data.

All authors read and approved the final manuscript.

## Note

Accession numbers of full genome sequences of hepatitis B virus in this manuscript.

HCC group 1

[DDBJ: AB670237, AB670238, AB670239, AB670240, AB670241, AB670242, AB670243, AB670244, AB670245, AB670246, AB670247, AB670248, AB670249, AB670250, AB670251, AB670252, AB670253, AB670254, AB670255, AB670256, AB670257, AB670258, AB670259, AB670260, AB670261, AB670262, AB670263, AB670264, AB670265, AB670266, AB670267, AB670268, AB670269, AB670270, AB670271, AB670272, AB670273]

non-HCC group 1

[DDBJ: AB670274, AB670275, AB670276, AB670277, AB670278, AB670279, AB670280, AB670281, AB670282, AB670283, AB670284, AB670285, AB670286, AB670287, AB670288, AB670289, AB670290, AB670291, AB670292, AB670293, AB670294, AB670295, AB670296, AB670297, AB670298, AB670299, AB670300, AB670301, AB670302, AB670303, AB670304, AB670305, AB670306, AB670307, AB670308, AB670309, AB670310, AB670311]

## Pre-publication history

The pre-publication history for this paper can be accessed here:

http://www.biomedcentral.com/1471-2407/11/458/prepub
